# Imaging c-Met expression using 18F-labeled binding peptide in human cancer xenografts

**DOI:** 10.1371/journal.pone.0199024

**Published:** 2018-06-12

**Authors:** Weihua Li, Hongqun Zheng, Jiankai Xu, Shaodong Cao, Xiuan Xu, Peng Xiao

**Affiliations:** 1 Department of Medical Imaging and Nuclear Medicine, the Fourth Affiliated Hospital, Harbin Medical University, Harbin, China; 2 Department of Surgical Oncology and Hepatobiliary Surgery, the Fourth Affiliated Hospital, Harbin Medical University, Harbin, China; 3 College of Bioinformatics Science and Technology, Harbin Medical University, Harbin, China; 4 New Iberia Research Center, University of Louisiana at Lafayette, New Iberia, Louisiana, United States of America; Wake Forest University School of Medicine, UNITED STATES

## Abstract

**Objectives:**

c-Met is a receptor tyrosine kinase shown inappropriate expression and actively involved in progression and metastasis in most types of human cancer. Development of c-Met-targeted imaging and therapeutic agents would be extremely useful. Previous studies reported that c-Met-binding peptide (Met-pep1, YLFSVHWPPLKA) specifically targets c-Met receptor. Here, we evaluated ^18^F-labeled Met-pep1 for PET imaging of c-Met positive tumor in human head and neck squamous cell carcinoma (HNSCC) xenografted mice.

**Methods:**

c-Met-binding peptide, Met-pep1, was synthesized and labeled with 4-nitrophenyl [^18^F]-2-fluoropropionate ([^18^F]-NPFP) ([^18^F]FP-Met-pep1). The cell uptake, internalization and efflux of [^18^F]FP-Met-pep1 were assessed in UM-SCC-22B cells. *In vivo* pharmacokinetics, blocking and biodistribution of the radiotracers were investigated in tumor-bearing nude mice by microPET imaging.

**Results:**

The radiolabeling yield for [^18^F]FP-Met-pep1 was over 55% with 97% purity. [^18^F]FP-Met-pep1 showed high tumor uptake in UM-SCC-22B tumor-bearing mice with clear visualization. The specificity of the imaging tracer was confirmed by significantly decreased tumor uptake after co-administration of unlabeled Met-pep1 peptides. Prominent uptake and rapid excretion of [^18^F]FP-Met-pep1 was also observed in the kidney, suggesting this tracer is mainly excreted through the renal-urinary routes. *Ex vivo* biodistribution showed similar results that were consistent with microPET imaging data.

**Conclusions:**

These results suggest that ^18^F-labeled c-Met peptide may potentially be used for imaging c-Met positive HNSCC cancer *in vivo* and for c-Met-targeted cancer therapy.

## Introduction

Mesenchymal-epithelial transition factor (c-Met) is a receptor tyrosine kinase for hepatocyte growth factor (HGF), and activation of c-Met can lead to tumor progression, metastasis and angiogenesis [1–3]. Studies have shown overexpression of c-Met linked to multiple human solid tumor types, including those of breast [4], ovary [5], lung [6], colon [7], pancreas [8], prostate [9], head and neck [10], stomach [11], liver [12], kidney [13], brain [14], and skin [15]. Thus, c-Met is considered to be a potential new target for developing therapeutic agents [16, 17], which would be extremely useful for diagnosing cancer by imaging c-Met expression and subsequently monitoring response to c-Met-targeted therapies [18].

Anti-c-Met monoclonal antibodies (mAb) were rapidly developed as nuclear imaging agents for treating various human cancers [19–21], but poor tumor penetration due to the big size of molecules, as well as liver or bone marrow toxicity, has limited applications [22]. Compared with mAb, peptides are considerably smaller (1–2 kDa) in size result in reaching their intended specific targets much faster. Also, peptides generally do not bind to the reticuloendothelial system, avoid eliciting strong immune responses upon repeated administration [23]. Therefore, peptides are promising carriers for delivering radionuclides into tumors for c-Met-directed imaging. Zhao *et al*. identified a c-Met binding peptide (Met-pep1, YLFSVHWPPLKA) targets explicitly c-Met receptor from a phage display of a combinatorial peptide library [24]. This peptide labeled with Iodine-125 reacted with c-Met on the cell surface and competed with the binding of HGF to c-Met. However, the image quality was not optimal using this tracer. Iodine-125, a low energy isotope with a 60-day half-life, makes it very attractive for *in vitro* radioimmunoassay and *ex vivo* autoradiography with high resolution, but its low energy also leads to low sensitivity for *in vivo* imaging with planar nuclear scintigraphy. Secondly, when the iodine is labeled on the tyrosine residue, deiodination can occur thus thyroid accumulation of radioactivity especially when the tracer is internalized inside the cells.

Several attempts are being made to improve image quality and quantification using various radioisotopes. Fluorine-18 [^18^F] is one of the most widely used positron emission isotopes for PET imaging because it has good imaging characteristics and ideally suited half-life (about 110 min) for the small-molecular-weight peptides [25–27]. So far, in a majority of cases, peptide labeling is achieved by using ^18^F-containing prosthetic groups [27–29]. However, ^18^F has not been used for the labeling of the Met-pep1 peptide for *in vivo* PET imaging in HNSCC model. Here, we have labeled the c-Met peptide (Met-pep1) with [^18^F]-NPFP and further evaluated the in vitro cell uptake, internalization, and efflux studies on UM-SCC-22B cells and *in vivo* distribution pattern and c-Met-targeting efficacy using microPET imaging in HNSCC xenograft-bearing mice.

## Materials and methods

### Protocol approval

All the experimental methods in the current study has been approved by the research committee at Harbin Medical University. All the experiments have been carried out in accordance with the guidelines from the research committee at Harbin Medical University. All animal experiments were approved by the Institutional Animal Care and Use Committee at Harbin Medical University. Surgeries were performed in accordance with the Principles of Laboratory Care, supervised by a qualified veterinarian.

### Reagents and chemicals

Fmoc protected amino acids were obtained from CS Bio Co. (Menlo Park, CA) and all other chemicals were purchased from Sigma-Aldrich (St. Louis, MO). Mass spectra (MS) were obtained on a Waters Acquity UPLC system coupled with Waters QT of Premier MS (LC-MS). ^1^H NMR spectra were recorded at 300 MHz on a Bruker 300 Ultra-Shield spectrometer in CDCl_3_ with tetramethylsilane (TMS) as the internal standard. For the purification of modified peptides, preparative reversed-phase HPLC was performed on Waters 600 gradient system with a Waters 996 Photodiode Array (PDA) detector using a Higgins PROTO 300 C18 column (5 μm, 250 × 20 mm). For purification of radiolabeled peptides, semi-prep reversed-phase HPLC was performed on a separate Waters 600 gradient system also with a 996 PDA detector plus a Beckman 170 radioisotope detector using Higgins PROTO 300 C_18_ column (5 μm, 250 × 10 mm). Analytical reversed-phase HPLC was run on a Perkin-Elmer Series 200 LC gradient system with a Waters 2784 Dual Absorbance UV detector plus a Bioscan radioisotope detector using a Waters Symmetry column (5 μm, 150 × 3.9 mm). The flow rate was 12 ml/min for the preparative column, 5ml/min for the semi-prep column and 1 ml/min for the analytical column running the same linear gradient starting from 5% A (0.1% TFA in acetonitrile) and 95% B (0.1% TFA in water) for 5 min and increasing A to 65% at 35 min. For the purification of radiolabeled prosthetic group, a separate Perkin Elmer Series 200 LC isocratic system was used with a Knauer 200 UV detector plus a Bioscan radioisotope detector using a Phenomenex Luna C18 column (5 μm, 250 × 10 mm). Waters C-18 Sep-Pak Cartridge was used for solid-phase extraction of the labeled prosthetic group and Varian BOND ELUT C_18_ column (50 mg) was used for solid-phase extraction of the labeled peptide. Fluoride-18 was obtained from NIH cyclotron facility.

### Synthesis of c-Met binding peptide

The c-Met binding peptide (NH_2_-Tyr-Leu-Phe-Ser-Val-His-Trp-Pro-Pro-Leu-Lys-Ala, Met-pep1) was synthesized on a peptide synthesizer and purified with preparative HPLC with a 25 min retention time. The identity of the peptide was confirmed with LC-MS. The peptide was collected and the solvents removed by lyophilization to afford white powders. LC-Ms of Met-pep1 (C_74_H_104_N_16_O_15_): [MH]^+^ = 1457.6571 (m/z), calc: 1456.7867.

### Preparation of 4-nitrophenyl [^18^F]-2-fluoropropionate ([^18^F]-NPFP)

The 4-nitrophenyl [^18^F]-2-fluoropropionate was prepared according to a published procedure with some modifications [30]. Briefly, 5 mg of ethyl 2-bromopropionate in 0.15 ml of acetonitrile was reacted with anhydrous [^18^F]fluoride containing 3.8 mg of K-222 and 0.7 mg of potassium carbonate to form ethyl 2-[^18^F]fluoropropionate. The radioactive ester was hydrolyzed with 0.15 ml of 0.2 N KOH and the solvent was blown to dryness to produce potassium salt of 2-[^18^F]fluoropropionic acid and was then converted to 4-nitrophenyl [^18^F]-2-fluoropropionate (NPFP) with 20 mg of bis-4-nitropenyl carbonate (BNPC) in 0.3 ml of acetonitrile. The final product was purified by HPLC on a semi-prep Phenomenex Luna C18 column. The desired product was collected (R_t_ = 29 min) and trapped on a Waters Sep-Pak Plus C18 cartridge and eluted with 1 ml CH_2_Cl_2_ to a plastic tube.

### Preparation of [^18^F]FP-Met-pep1

The CH_2_Cl_2_ in the tube containing [^18^F]NPFP was removed with argon flow at room temperature and 1.0 mg of Met-pep1 in 0.1 ml of DMSO containing 20 μl of DIPEA was added to the tube and heated at 80°C for 10 min. The reaction mixture was cooled and diluted with 0.7 ml of water containing 25 μl of acetic acid and injected onto a semi-prep HPLC column (Vydac C18) running a linear gradient starting from 5% A (0.1% TFA in acetonitrile) and 95% B (0.1% in water) for 2 min and increasing A to 65% at 32 min at 5 ml/min. The radioactive peak at retention time of 23.1 min was collected and added 10 ml of water and trapped on a Varian Bond Elut C18 column (100 mg). The radioactivity trapped on the C18 column was eluted with 0.3 ml of 1 mM HCl ethanol solution and the solvent was evaporated with argon flow and the product was re-dissolved in normal saline for further use.

### Cell lines and tumor models

The human head and neck squamous carcinoma (HNSCC) cell line UM-SCC-22B (obtained from the University of Michigan) was used in this study. The cells were maintained in DMEM supplemented with 10% fetal bovine serum (FBS), 1% glutamine, 100 unit/ml penicillin, and 100 mg/ml streptomycin (Invitrogen, CA). Subcutaneous UM-SCC-22B tumor model was established in 4 to 6 weeks old male athymic *nu/nu* mice (Harlan Laboratories). Typically, 5 × 10^6^ cells suspended in 50 μl of PBS were injected in the right or left front shoulder. Tumor growth was followed by caliper measurements of perpendicular diameters of the tumor. The tumor volume was estimated by the formula: tumor volume = a×b^2^ /2, where a and b were the tumor length and width, respectively, in millimeters. Small animal PET studies were performed when the tumor volume reached 100 to 200 mm^3^.

### Cell uptake, internalization and efflux studies

For cell uptake measurement, UM-SCC-22B cells were seeded into a 24-well plate at a density of 1×10^5^ cells per well and incubated with 18.5 kBq (0.5 μCi)/well of [^18^F]FP-Met-pep1 at 37°C for 15, 30 and 60 min. Tumor cells were then washed twice with cold PBS and harvested by adding 250 μl of 0.1 N NaOH. Internalization assay was preformed similarly to the procedure described above. After 15, 30 and 60 min incubation of UM-SCC-22B cells with [^18^F]FP-Met-pep1 at 37°C, the cells were washed twice with cold PBS and then incubated for 1 min with acid-washing-buffer (50 mM glycine, 0.1 M NaCl, pH = 2.8) to remove surface bound radioactive ligand. Thereafter, the cells were washed twice with cold PBS and harvested by adding 250 μl of 0.1 N NaOH. The blocking assay was preformed similarly to the procedure described above except that the unlabeled c-Met binding peptide was added into the wells (500 μg/well) and incubated for 15 min at 37°C before adding [^18^F]FP-Met-pep1. For efflux experiment, all UM-SCC-22B cells in a 24-well plate were initially incubated with 18.5 kBq (0.5 μCi)/well of [^18^F]FP-Met-pep1 at 37°C for 1 hour. The cells were then washed twice with cold PBS, and incubated with serum free DMEM medium for 15, 30 and 60 min. At each time point, the cells were washed twice with PBS, and harvested by adding 250 μl of 0.1 N NaOH. The cell suspensions were collected and measured in a gamma counter and the cell uptake, internalization and efflux were expressed as the percentage of the added dose (%AD) after decay correction to EOB. Each data point is an average of triplicate wells.

### MicroPET imaging analysis

To perform a microCT scan by a high-resolution Inveon MicroCT scanner (Siemens Medical Solutions) before microPET scan, an anesthetized nude mouse bearing UM-SCC-22B tumor was mounted on a turntable bed that could be moved automatically in the axial direction. Whole body CT acquisition parameters were as follows: voltage 70 kVp, current 400 μA, angular sampling 1° per projection for a full 360° scan, and effective pixel size 58 μm. Scan time was approximately 12 min. The image was reconstructed in real-time by a modified Feldkamp cone bean algorithm with a Shepp-Logan filter and appropriate center offset determined prior to scanning. PET scans were performed using an Inveon microPET scanner (Siemens Medical Solutions). Tumor-bearing mice were anesthetized using isoflurane/O_2_ (1.5–2% v/v) and intravenously injected with 3.7 MBq (100 μCi) of [^18^F]FP-Met-pep1 in a volume of 100 μl saline. For blocking experiments, [^18^F]FP-Met-pep1 was co-injected with 500 μg of unlabeled Met-pep1 peptide. Each group contained four mice. The images were reconstructed by a three-dimensional ordered subsets expectation maximization (3D-OSEM) algorithm with maximum a posteriori (MAP) algorithm and the frame rates were 10 × 30s, 5 × 60s, 5 × 120s and 10 × 240s, and no correction was applied for attenuation or scatter. Static images at 1 h time point were also acquired as 10-min static images for region of interest (ROI) quantification. Image analysis was done using ASI Pro VMTM software. PET/CT images were co-registered and fused automatically with ASI software. For each microPET scan, three-dimensional ROIs were drawn over the tumor, liver, kidney, and muscle on decay-corrected whole-body coronal images. The average radioactivity concentration within a tumor or an organ was obtained from mean pixel values within the ROI volume, which were converted to counts per milliliter by using a pre-determined conversion factor.

### Biodistribution studies in tumor-bearing mice

After microPET imaging, the blood of mice bearing UM-SCC-22B tumor xenografts was drawn from the orbital sinus, then mice were sacrificed by cervical dislocation and selected organs were excised. Blood, tumor, and major organs tissues were collected and weighed (wet weight). The radioactivity in each tissue was measured using a gamma-counter (Packard Instrument). Distribution of activity was calculated as percentage of injected dose per gram of organ (%ID/g). For each mouse, the radioactivity of the tissue samples was calibrated against a known aliquot of the injected dose and normalized to a body mass of 30 g. Values were expressed as mean ± SD for a group of four animals.

### Statistical analysis

Quantitative data are expressed as mean ± SD. The One-way ANOVA and Two-tailed Unpaired t test, confidence interval of 95% (95% CI), were used to analyze group differences. Results of statistical analyses were considered significant if P values of ≤ 0.05.

## Results

### Radiosynthesis

**Fig 1:**
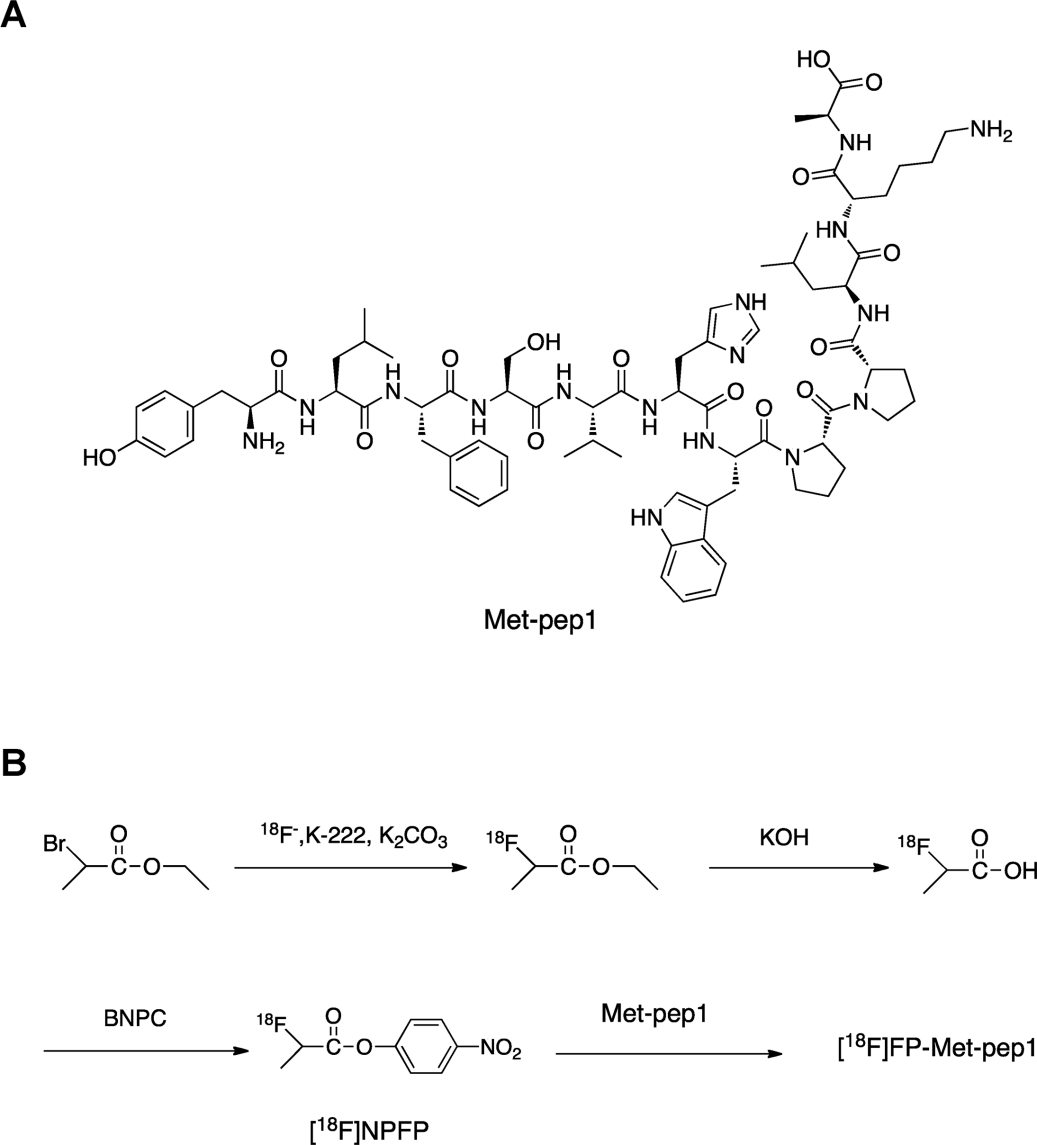
(A) Chemical structures of c-Met binding peptide, Met-pep1. (B) Radiosynthesis of [^18^F]FP-Met-pep1.

The c-Met binding peptide, Met-pep1 (**Fig 1A)** was first prepared on a peptide synthesizer with 98% purity. Radiosynthesis of [^18^F]FP-Met-pep1 is shown in **Fig 1B**. Total of 8 runs were performed for [18F]NPFP and specific activity were about 55.5 GBq/nmol (1500 mCi/nmol) at the end of bombardment (EOB). The radiochemical yield for [^18^F]FP-Met-pep1 was over 55% with specific activity of 7.4 GBq/nmol (200 mCi/nmol). The radiochemical purity of [^18^F]FP-Met-pep1 was over 97% by analytical HPLC analysis.

### Uptake, internalization and efflux

**Fig 2:**
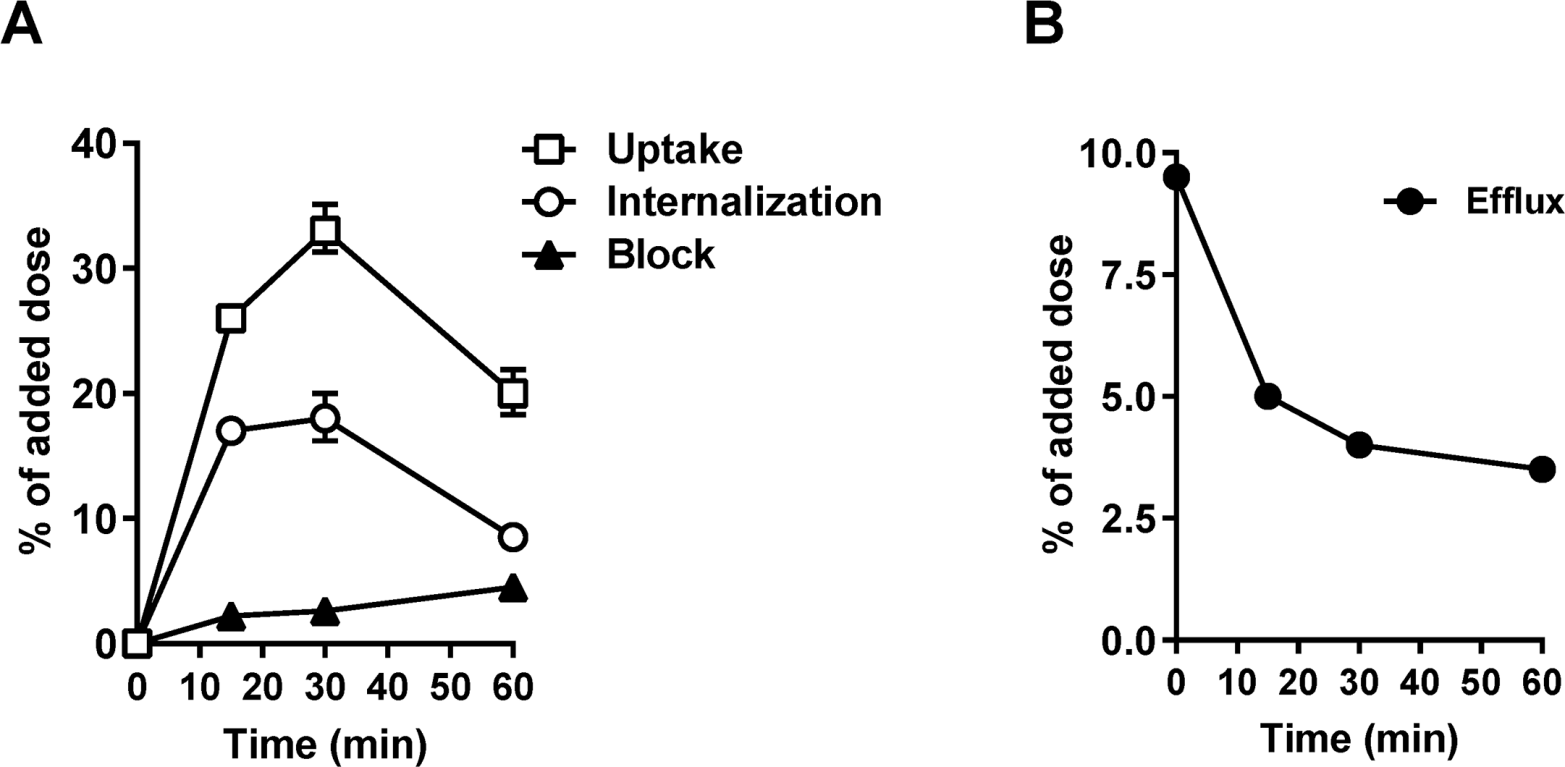
Cell uptake, internalization and blocking assays (A) and efflux (B) of [^18^F]FP-Met-pep1 in UM-SCC-22B cells. Data are from two experiments with triplicate samples and are expressed as mean ± SD.

The cell uptake, internalization and efflux of [^18^F]FP-Met-pep1 were evaluated in cell line UM-SCC-22B [31], which is a head and neck squamous carcinoma cell line overexpressing human c-Met. It was established from the metastatic lymph node in the neck of a female patient and is a suitable *in vitro* model of H&N tumor studies. As shown in **Fig 2**, high level of cell uptake for [^18^F]FP-Met-pep1was observed, exhibiting a rapid increase at first 15 min, reaching its peak at 30 min (34.52 ± 1.34%) and decreasing at 60 min (**Fig 2A**). Similarly, [^18^F]FP-Met-pep1 also showed a high level of internalization after 15, 30 and 60 min incubation, respectively. Zhao et al study have proved that Met-pep1 internalizes cells via receptor specific binding followed by endocytosis. Approximately 2/3 of the observed uptake was due to the internalization of [^18^F]FP-Met-pep1 into the cells in the early phase, which was decreased to about 1/3 at 60 min. In contrast, dramatic inhibition of cell uptake was observed in the presence of 500 μg of unlabeled Met-pep1 peptide (**Fig 2A**), indicating specific cellular binding and prominent uptake of [^18^F]FP-Met-pep1 in c-Met positive tumor cells. When the labeled cells were incubated in serum-free medium devoid of radioactivity, the [^18^F]FP-Met-pep1 showed dissociation and efflux from the cells during the time (**Fig 2B**). During 15 min, approximately 47% of [^18^F]FP-Met-pep1 had effluxed out of the UM-SCC-22B cells. The efflux rate of [^18^F]FP-Met-pep1 became slower after 30 min. At the end of incubation, about 36% of the radiotracer remained bound with the cells.

### MicroPET imaging

**Fig 3:**
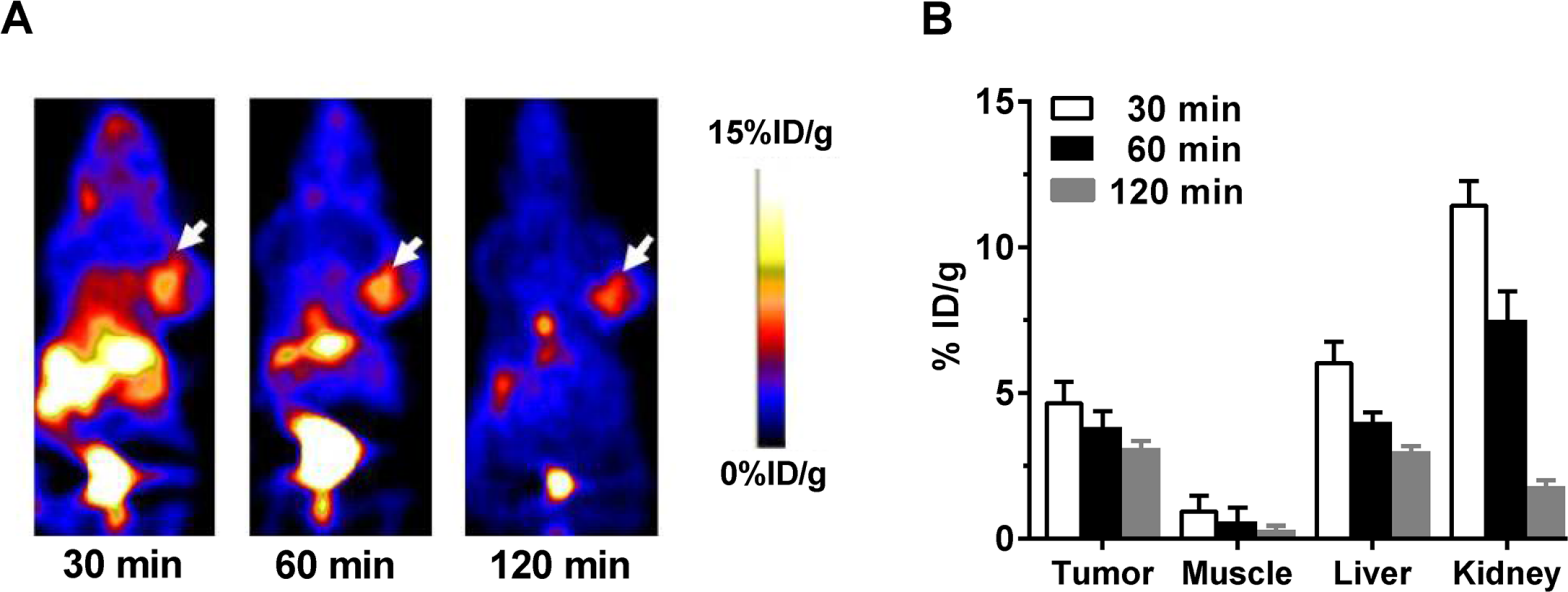
*In vivo* PET imaging of UM-SCC-22B xenografted mice by [^18^F]FP-Met-pep1. (A) Decay-corrected whole-body coronal microPET images of UM-SCC-22B tumor-bearing mice at 30, 60 and 120 min after injection of 3.7 MBq (100 μCi) of [^18^F]FP-Met-pep1. Arrows indicate tumor on right shoulder. (B) Quantification of [^18^F]FP-Met-pep1 uptake in UM-SCC-22B tumor, muscle, liver and kidney (n = 4 per group). Data are presented as mean ± SD.

To assess tumor-targeting efficiency, microPET imaging study was performed *in vivo* in a UM-SCC-22B tumor-bearing mice model developed by subcutaneous inoculation. Representative coronal images at different time points after intravenous administration of [^18^F]FP-Met-pep1 are shown in **Fig 3A** and **S1 Fig**. The UM-SCC-22B tumors were clearly visible in relation to the contralateral background with uptakes of 4.72 ± 0.67, 3.83 ± 0.55 and 3.11 ± 0.25%ID/g at 30, 60 and 120 min post injection, respectively (**Fig 3B**). Both kidney and liver showed higher uptake at 30 min post-injection with %ID/g of 6.15 ± 0.71 and 11.5 ± 0.78 respectively, but the tracer was washed out during time (**Fig 3B**). There was rapid clearance and excretion of radioactivity, primarily through the renal pathway, with about 65% excreted within 2 h after injection (**S1 Fig**), suggesting that it is mainly excreted through the renal-urinary routes. In contrast, muscle had very low uptake during the all timepoints.

**Fig 4:**
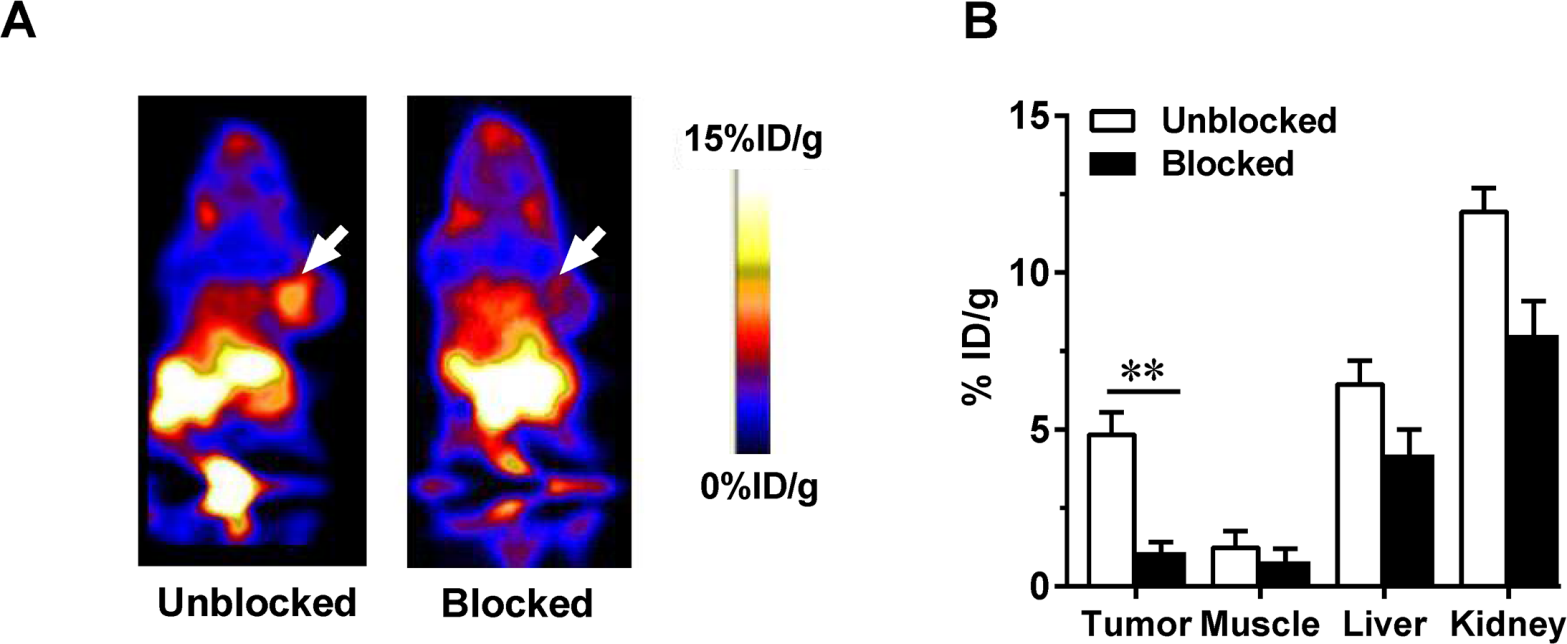
(A) Representative coronal PET images of UM-SCC-22B tumor-bearing mice (right shoulder) (B) Quantification of [^18^F]FP-Met-pep1 in UM-SCC-22B tumor, muscle, liver, and kidney at 30 min after injection with 3.7 MBq (100 μCi) of [^18^F]FP-Met-pep1 in the absence or presence of 500 μg of unlabeled c-Met binding peptide. ROIs are shown as mean %ID/g ± SD. The level of significance is indicated by p-values as follows: * p<0.05; ** p<0.01.

The receptor specificity of the tracer accumulation was confirmed by a blocking assay, in which 500 μg of unlabeled Met-pep1 peptide was injected 30 min before the tracer injection (**Fig 4A**). As shown in **Fig 4B**, in the absence or presence of 500 μg of unlabeled Met-pep1 peptide, the tumor uptake of [^18^F]FP-Met-pep1 was significantly lower (95% CI; p = 0.0023) than that of unblocked tumors (1.15 ± 0.25 vs. 4.85 ± 0.66, n = 4). Note that reduced uptakes were also observed in other organs and tissues in the presence of blocking dose of c-Met binding peptide, but the differences in normal organs were less marked than that in the tumor.

### Biodistribution

**Fig 5:**
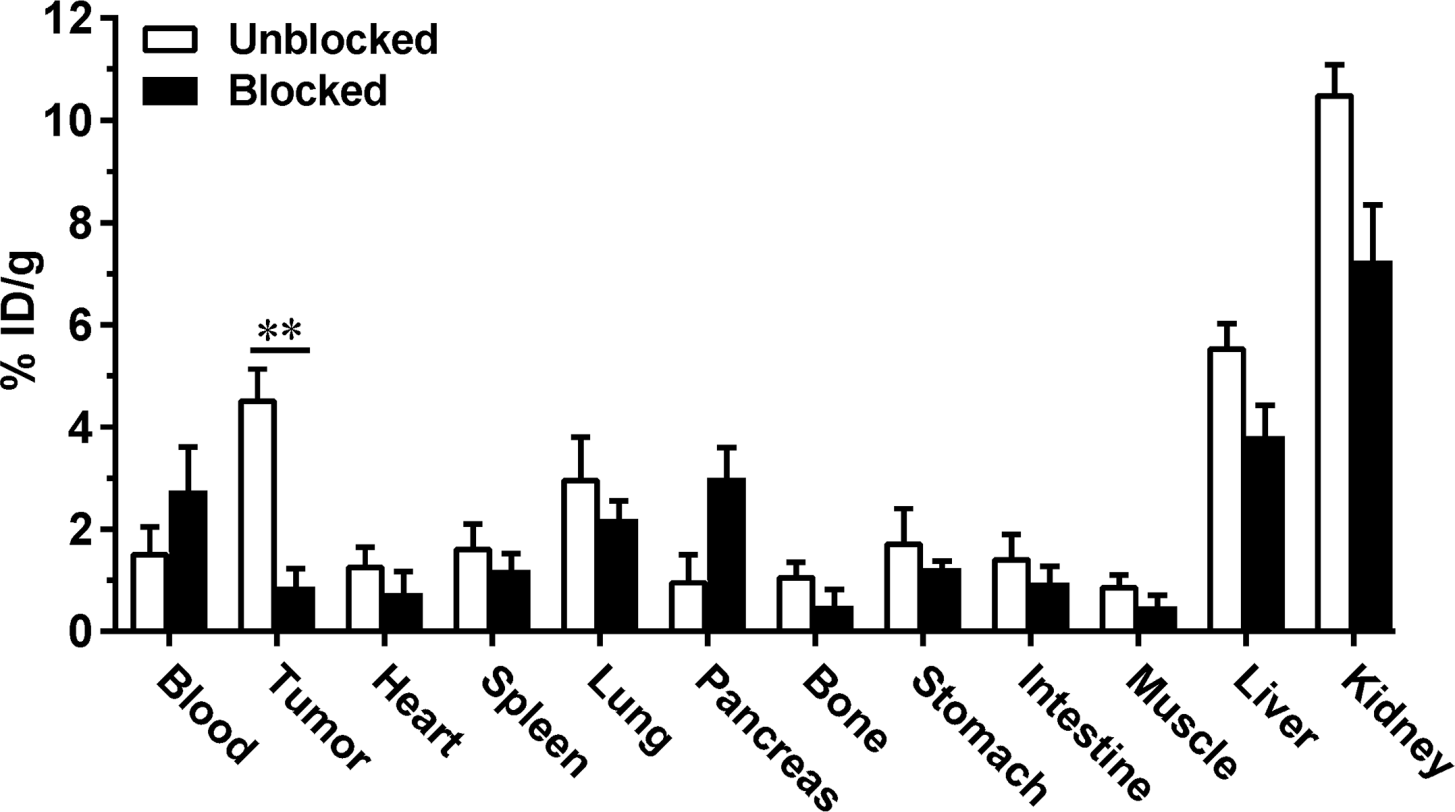
*Ex vivo* biodistribution of [^18^F]FP-Met-pep1 (3.7 MBq) in UM-SCC-22B tumor-bearing nude mice at 30 min after microPET scans with or without 500 μg of unlabeled c-Met binding peptide as blocking agent. Data are presented as mean %ID/g ± SD. The level of significance is indicated by p-values as follows: * p<0.05; ** p<0.01.

To further confirm the microPET imaging quantification, the biodistribution of [^18^F]FP-Met-pep1 was assessed in UM-SCC-22B tumor-bearing athymic nude mice immediately after microPET imaging. As shown in **Fig 5**, the tumor uptakes measured by direct tissue sampling and gamma-counting were 4.58 ± 0.59 and 0.88 ± 0.35%ID/g at 30 min after injection in the absence or presence of 500 μg unlabeled peptide, which was consistent with PET imaging data (95% CI; p = 0.0019). The circulation time of [^18^F]FP-Met-pep1 was longer since blood tracer concentration increased from 1.55 ± 0.48 to 2.85 ± 0.76%ID/g after blocking. Interestingly, the uptake in the pancreas was also increased after unlabeled peptide blocking. Similarly, the tracer accumulations in the kidney and liver were much higher (10.54 ± 0.53 and 5.58 ± 0.45%ID/g, respectively) and even after the peptide blocking. There was no significant change of uptake in other organs, including heart, lung, spleen, bone marrow, stomach and intestine.

## Discussion

The c-Met is a membrane-spanning receptor tyrosine kinase (RTK) whose ligand is the hepatocyte growth factor (HGF). Its aberrant activity is documented in HNSCC as well as many other cancers. Over three decades of research have been spent investigating the HGF/c-Met pathway and its clinical relevance. Overexpression of c-Met is commonly associated with poorer outcomes in patients. Notably, c-Met contributes to treatment resistance by bypassing traditionally clinically inhibited signals such as EGF [31]. Further, there is a need to develop c-Met-specific agents and use these in patients with appropriate biomarkers.

There are several tested preclinical c-Met PET tracers, including ^64^Cu-labeled HGF protein [32], ^89^Zr-labeled anticalin PRS-110 [33], ^89^Zr-labeled full-length antibody DN30 [21] and 1-armed antibody ^89^Zr-Ornartuzumab [34]. They have different properties, such as binding site, residualizing radiometals for improved contrast and different tumor targeting kinetics, and clearance rates. However, optimal imaging time points were estimated to be between two and five days after tracer administration, thus hampering potential routine clinical use as diagnostic agents. In contrast, the biodistribution of small c-Met peptides, such as ^18^F-AH113804 [35], permitted early imaging after tracer administration as the diagnostic agents. ^18^F-AH113804, a peptide-based molecular imaging agent for human c-Met, has been used for detection of early-stage locoregional recurrence in a basal-like breast cancer model [35]. Further, a phase-1 biodistribution of ^18^F-AH113804 study confirmed the clinical safety in healthy adult volunteers [36]. Next to c-Met imaging with a PET tracer, imaging with a fluorescent tracer might be of interest. GE-137, a fluorescent c-Met probe of which the targeting moiety is based on the same peptide of AH113804, was successfully applied to detect polyps in individuals at risk for colorectal cancer [37]. Benefits of this strategy compared with radionuclide-based imaging are a less costly infrastructure and availability of off the shelf nonradioactive tracers. It is especially of interest when information of a limited body area is needed because no whole-body information can be obtained.

To the best of our knowledge, imaging of c-Met peptide in human H&N cancer model has not been reported to date. The peptide we chose was based on the study by Zhao *et al*. who obtained the peptide bind specifically to receptor c-Met from a phage display library [24]. They have demonstrated that this peptide binds to c-Met specifically with high affinity by some *in vitro* assays. They also performed nuclear imaging of the radioiodinated peptide in a mouse xenograft model and showed tumor-associated activity, recommending this peptide as a promising candidate for future clinical applications. Although radioiodinated receptor-binding peptides already represent an important class of radiopharmaceuticals, the image quality was not reached optimal levels. Despite the fact that this c-Met peptide has a tyrosine amino acid residue where is for radioiodination, it also contains a lysine serves as an anchor residue for ^18^F based radiolabeling.

In this study, we achieved site-specific labeling of c-Met peptide with ^18^F and demonstrated by microPET imaging that the ^18^F- labeled tracer was able to accumulate in c-Met positive HNSCC tumors. The 4-nitrophenyl [^18^F]-2-fluoropropionate ([^18^F]-NPFP) was chosen as the labeling agent due to its small size that will have minimal disturbance on the affinity of the peptide. The peptide also has an α-amine on tyrosine residue which could also be labeled, however, the reactivity is much lower than the amine on lysine and the majority of the label is attached on the lysine. After labeling with [^18^F]-NPFP, a similar time-dependent cellular uptake and internalization were seen for [^18^F]FP-Met-pep1 in c-Met positive UM-SCC-22B cells, and about 54% of the radioactivity uptake was internalized within 30 min by UM-SCC-22B cells. Despite tumor uptake dropped rapidly at 60 min, 45% of radioactivity uptake (internalization) was still retained with the cells (**Fig 2A**). Similar patterns were observed *in vivo* imaging study, except for two mice at 120 min post-injection in which the tumors became much less visible (**Panels B and D in S2 Fig**). We speculate that the signal decrease was primarily due to rapid clearance from the bloodstream and excretion via the renal-urinary pathway. We also confirmed that tumor uptake is specific since the rapid cellular uptake of the tracer could be effectively blocked by the extra amount of unlabeled peptides. In PET imaging study, the tumors are clearly visualized at early time points with high contrast after intravenous injection of [^18^F]FP-Met-pep1 and the image quality is much better than that those reported in the literature obtained by planar nuclear scintigraphy. The main route of the tracer clearance was through kidney and it was verified by the time-activity curves which showed fast uptake and moderate clearance of [^18^F]FP-Met-pep1 through renal-urinary routes leading to good target to background radio. The clearance may be caused peptide degradation inside cells and subsequent efflux of radiolabeled fragments. In our ^18^F-labeled c-Met binding peptide, it is not a major concern since the radiolabeled fragments can be cleared through the kidneys, but this could be a major issue for ^125^I-labeled peptide due to the release of radioactive iodide. In agreement with the PET imaging study, the *ex vivo* biodistribution results further validated higher tumor uptake and lower background.

In this study, we chose a novel, peptide-based molecular imaging agent with ^18^F-tracer, which binds to human c-Met with high affinity, has a favorable kinetic profile, exhibits specific uptake in c-Met positive H&N tumor mouse model, and rapid renal clearance. However, certain limitations are inevitable in our study: 1) we only reported the ^18^F-c-Met peptide imaging results in male mice not in females, gender-specific differences in effective dose and biodistribution might occur; 2) we haven’t tested whether it can differentiate between high and low c-Met-expressing HNSCC; 3) we haven’t tested the usage in other types of tumor models. These limitations point to the need for further research and that will be considered in future investigations.

It is worth pointing out that c-Met also plays a major role in compensating for inhibition of RTK pathways that drive proliferation and metastasis in many tumors including HNSCC tumor. Our ^18^F-labeled c-Met peptide specifically binds to c-Met-expressing tumor cells *in vitro* makes it possible to visualize the abnormal alteration of c-Met expression *in vivo* and in real time. Targeting c-Met in conjunction with these pathways may lead to more effective therapeutic strategies and stronger treatment modalities in the near future. On further optimization and development, [^18^F]FP-Met-pep1 may be translated into the clinic for more applications such as patient screening for c-Met-targeted therapeutics, monitoring therapeutic effects, evaluating prognosis, and analysis for resistance mechanism.

## Supporting information

S1 Fig (A) Dynamic decay-corrected whole-body coronal microPET images after injection of 3.7 MBq (100 μCi) of [^18^F]FP-Met-pep1 in UM-SCC-22B tumor–bearing mice. (B) Time-activity curves of [^18^F]FP-Met-pep1 in UM-SCC-22B tumor, blood, liver, kidney and muscle.
(TIFF)

S2 Fig *In vivo* microPET images of four tumor-bearing mice (A)—(D) at 30, 60 and 120 min after injection of 3.7 MBq (100 μCi) of [^18^F]FP-Met-pep1. Tumors are indicated by arrows.
(TIFF)

## References

[1] Takayama H, LaRochelle WJ, Sharp R, Otsuka T, Kriebel P, Anver M, et al Diverse tumorigenesis associated with aberrant development in mice overexpressing hepatocyte growth factor/scatter factor. Proc Natl Acad Sci U S A. 1997;94(2):701–6. ; PubMed Central PMCID: PMCPMC19577. 9012848 10.1073/pnas.94.2.701 PMC19577

[2] Birchmeier C, Birchmeier W, Gherardi E, Vande Woude GF. Met, metastasis, motility and more. Nat Rev Mol Cell Biol. 2003;4(12):915–25. doi: 10.1038/nrm1261 . 14685170 10.1038/nrm1261

[3] Boccaccio C, Comoglio PM. Invasive growth: a MET-driven genetic programme for cancer and stem cells. Nat Rev Cancer. 2006;6(8):637–45. doi: 10.1038/nrc1912 . 16862193 10.1038/nrc1912

[4] Ponzo MG, Lesurf R, Petkiewicz S, O'Malley FP, Pinnaduwage D, Andrulis IL, et al Met induces mammary tumors with diverse histologies and is associated with poor outcome and human basal breast cancer. Proc Natl Acad Sci U S A. 2009;106(31):12903–8. doi: 10.1073/pnas.0810402106 ; PubMed Central PMCID: PMCPMC2722321. 19617568 10.1073/pnas.0810402106 PMC2722321

[5] Huntsman D, Resau JH, Klineberg E, Auersperg N. Comparison of c-met expression in ovarian epithelial tumors and normal epithelia of the female reproductive tract by quantitative laser scan microscopy. Am J Pathol. 1999;155(2):343–8. doi: 10.1016/S0002-9440(10)65130-9 ; PubMed Central PMCID: PMCPMC1866871. 10433927 10.1016/S0002-9440(10)65130-9 PMC1866871

[6] Ma PC, Kijima T, Maulik G, Fox EA, Sattler M, Griffin JD, et al c-MET mutational analysis in small cell lung cancer: novel juxtamembrane domain mutations regulating cytoskeletal functions. Cancer Res. 2003;63(19):6272–81. . 14559814

[7] Herynk MH, Radinsky R. The coordinated functional expression of epidermal growth factor receptor and c-Met in colorectal carcinoma metastasis. In Vivo. 2000;14(5):587–96. . 11125542

[8] Ebert M, Yokoyama M, Friess H, Buchler MW, Korc M. Coexpression of the c-met proto-oncogene and hepatocyte growth factor in human pancreatic cancer. Cancer Res. 1994;54(22):5775–8. . 7954397

[9] Humphrey PA, Zhu X, Zarnegar R, Swanson PE, Ratliff TL, Vollmer RT, et al Hepatocyte growth factor and its receptor (c-MET) in prostatic carcinoma. Am J Pathol. 1995;147(2):386–96. ; PubMed Central PMCID: PMCPMC1869824. 7639332 PMC1869824

[10] Lau PC, Chan AT. Novel therapeutic target for head and neck squamous cell carcinoma: HGF-MET signaling pathway. Anticancer Drugs. 2011;22(7):665–73. Epub 2011/06/29. doi: 10.1097/CAD.0b013e328341879d . 21709616 10.1097/CAD.0b013e328341879d

[11] Zhao J, Zhang X, Xin Y. Up-regulated expression of Ezrin and c-Met proteins are related to the metastasis and prognosis of gastric carcinomas. Histol Histopathol. 2011;26(9):1111–20. Epub 2011/07/14. doi: 10.14670/HH-26.1111 . 21751142 10.14670/HH-26.1111

[12] Ueki T, Fujimoto J, Suzuki T, Yamamoto H, Okamoto E. Expression of hepatocyte growth factor and its receptor c-met proto-oncogene in hepatocellular carcinoma. Hepatology. 1997;25(4):862–6. doi: 10.1002/hep.510250413 . 9096589 10.1002/hep.510250413

[13] Schmidt L, Duh FM, Chen F, Kishida T, Glenn G, Choyke P, et al Germline and somatic mutations in the tyrosine kinase domain of the MET proto-oncogene in papillary renal carcinomas. Nat Genet. 1997;16(1):68–73. doi: 10.1038/ng0597-68 . 9140397 10.1038/ng0597-68

[14] Jung W, Castren E, Odenthal M, Vande Woude GF, Ishii T, Dienes HP, et al Expression and functional interaction of hepatocyte growth factor-scatter factor and its receptor c-met in mammalian brain. J Cell Biol. 1994;126(2):485–94. ; PubMed Central PMCID: PMCPMC2200035. 8034747 10.1083/jcb.126.2.485 PMC2200035

[15] Saitoh K, Takahashi H, Sawada N, Parsons PG. Detection of the c-met proto-oncogene product in normal skin and tumours of melanocytic origin. J Pathol. 1994;174(3):191–9. doi: 10.1002/path.1711740308 . 7823252 10.1002/path.1711740308

[16] Puri N, Ahmed S, Janamanchi V, Tretiakova M, Zumba O, Krausz T, et al c-Met is a potentially new therapeutic target for treatment of human melanoma. Clin Cancer Res. 2007;13(7):2246–53. doi: 10.1158/1078-0432.CCR-06-0776 . 17404109 10.1158/1078-0432.CCR-06-0776

[17] Christensen JG, Burrows J, Salgia R. c-Met as a target for human cancer and characterization of inhibitors for therapeutic intervention. Cancer Lett. 2005;225(1):1–26. doi: 10.1016/j.canlet.2004.09.044 ISI:000229850600001. 15922853 10.1016/j.canlet.2004.09.044

[18] Hay R, Cao B, Tsarfaty I, Tsarfaty G, Resau J, Vande Woude G. Grappling with metastatic risk: bringing molecular imaging of Met expression toward clinical use. J Cell Biochem Suppl. 2002;39:184–93. doi: 10.1002/jcb.10441 . 12552618 10.1002/jcb.10441

[19] Hay RV, Cao B, Skinner RS, Wang LM, Su Y, Resau JH, et al Radioimmunoscintigraphy of human met-expressing tumor xenografts using met3, a new monoclonal antibody. Clin Cancer Res. 2003;9(10 Pt 2):3839S–44S. . 14506181

[20] Hay RV, Cao B, Skinner RS, Su Y, Zhao P, Gustafson MF, et al Nuclear imaging of Met-expressing human and canine cancer xenografts with radiolabeled monoclonal antibodies (MetSeek). Clin Cancer Res. 2005;11(19 Pt 2):7064s–9s. doi: 10.1158/1078-0432.CCR-1004-0014 . 16203803 10.1158/1078-0432.CCR-1004-0014

[21] Perk LR, Stigter-van Walsum M, Visser GW, Kloet RW, Vosjan MJ, Leemans CR, et al Quantitative PET imaging of Met-expressing human cancer xenografts with 89Zr-labelled monoclonal antibody DN30. Eur J Nucl Med Mol Imaging. 2008;35(10):1857–67. doi: 10.1007/s00259-008-0774-5 . 18491091 10.1007/s00259-008-0774-5

[22] Reilly RM, Sandhu J, Alvarez-Diez TM, Gallinger S, Kirsh J, Stern H. Problems of delivery of monoclonal antibodies. Pharmaceutical and pharmacokinetic solutions. Clin Pharmacokinet. 1995;28(2):126–42. doi: 10.2165/00003088-199528020-00004 . 7736688 10.2165/00003088-199528020-00004

[23] Aina OH, Sroka TC, Chen ML, Lam KS. Therapeutic cancer targeting peptides. Biopolymers. 2002;66(3):184–99. doi: 10.1002/bip.10257 . 12385037 10.1002/bip.10257

[24] Zhao P, Grabinski T, Gao C, Skinner RS, Giambernardi T, Su Y, et al Identification of a met-binding peptide from a phage display library. Clin Cancer Res. 2007;13(20):6049–55. doi: 10.1158/1078-0432.CCR-07-0035 . 17947467 10.1158/1078-0432.CCR-07-0035

[25] Teare H, Robins EG, Kirjavainen A, Forsback S, Sandford G, Solin O, et al Radiosynthesis and evaluation of [18F]Selectfluor bis(triflate). Angew Chem Int Ed Engl. 2010;49(38):6821–4. doi: 10.1002/anie.201002310 . 20715033 10.1002/anie.201002310

[26] Miller PW, Long NJ, Vilar R, Gee AD. Synthesis of 11C, 18F, 15O, and 13N radiolabels for positron emission tomography. Angew Chem Int Ed Engl. 2008;47(47):8998–9033. doi: 10.1002/anie.200800222 . 18988199 10.1002/anie.200800222

[27] Olberg DE, Hjelstuen OK. Labeling strategies of peptides with (1)(8)F for positron emission tomography. Curr Top Med Chem. 2010;10(16):1669–79. . 20583991 10.2174/156802610793176747

[28] Poethko T, Schottelius M, Thumshirn G, Hersel U, Herz M, Henriksen G, et al Two-step methodology for high-yield routine radiohalogenation of peptides: (18)F-labeled RGD and octreotide analogs. J Nucl Med. 2004;45(5):892–902. . 15136641

[29] Li W, Niu G, Lang L, Guo N, Ma Y, Kiesewetter DO, et al PET imaging of EGF receptors using [18F]FBEM-EGF in a head and neck squamous cell carcinoma model. Eur J Nucl Med Mol Imaging. 2012;39(2):300–8. doi: 10.1007/s00259-011-1969-8 ; PubMed Central PMCID: PMCPMC3262064. 22109665 10.1007/s00259-011-1969-8 PMC3262064

[30] Yang M, Gao H, Zhou Y, Ma Y, Quan Q, Lang L, et al F-Labeled GRPR Agonists and Antagonists: A Comparative Study in Prostate Cancer Imaging. Theranostics. 2011;1:220–9. ; PubMed Central PMCID: PMCPMC3086613. 21544226 10.7150/thno/v01p0220 PMC3086613

[31] Xu H, Stabile LP, Gubish CT, Gooding WE, Grandis JR, Siegfried JM. Dual blockade of EGFR and c-Met abrogates redundant signaling and proliferation in head and neck carcinoma cells. Clin Cancer Res. 2011;17(13):4425–38. doi: 10.1158/1078-0432.CCR-10-3339 ; PubMed Central PMCID: PMCPMC3138116. 21622718 10.1158/1078-0432.CCR-10-3339 PMC3138116

[32] Luo H, Hong H, Slater MR, Graves SA, Shi S, Yang Y, et al PET of c-Met in Cancer with (6)(4)Cu-Labeled Hepatocyte Growth Factor. J Nucl Med. 2015;56(5):758–63. doi: 10.2967/jnumed.115.154690 ; PubMed Central PMCID: PMCPMC4417426. 25840981 10.2967/jnumed.115.154690 PMC4417426

[33] Terwisscha van Scheltinga AG, Lub-de Hooge MN, Hinner MJ, Verheijen RB, Allersdorfer A, Hulsmeyer M, et al In vivo visualization of MET tumor expression and anticalin biodistribution with the MET-specific anticalin 89Zr-PRS-110 PET tracer. J Nucl Med. 2014;55(4):665–71. doi: 10.2967/jnumed.113.124941 . 24614223 10.2967/jnumed.113.124941

[34] Jagoda EM, Lang L, Bhadrasetty V, Histed S, Williams M, Kramer-Marek G, et al Immuno-PET of the hepatocyte growth factor receptor Met using the 1-armed antibody onartuzumab. J Nucl Med. 2012;53(10):1592–600. doi: 10.2967/jnumed.111.102293 ; PubMed Central PMCID: PMCPMC3982858. 22917884 10.2967/jnumed.111.102293 PMC3982858

[35] Arulappu A, Battle M, Eisenblaetter M, McRobbie G, Khan I, Monypenny J, et al c-Met PET Imaging Detects Early-Stage Locoregional Recurrence of Basal-Like Breast Cancer. J Nucl Med. 2016;57(5):765–70. doi: 10.2967/jnumed.115.164384 . 26635342 10.2967/jnumed.115.164384

[36] Somer EJ, Owenius R, Wall A, Antoni G, Thibblin A, Sorensen J. The clinical safety, biodistribution and internal radiation dosimetry of [(18)F]AH113804 in healthy adult volunteers. EJNMMI Res. 2016;6(1):87 doi: 10.1186/s13550-016-0239-y ; PubMed Central PMCID: PMCPMC5126032. 27896673 10.1186/s13550-016-0239-y PMC5126032

[37] Burggraaf J, Kamerling IM, Gordon PB, Schrier L, de Kam ML, Kales AJ, et al Detection of colorectal polyps in humans using an intravenously administered fluorescent peptide targeted against c-Met. Nat Med. 2015;21(8):955–61. doi: 10.1038/nm.3641 . 26168295 10.1038/nm.3641

