# Supplementary figures and images for: Imaging c-Met expression using 18F-labeled binding peptide in human cancer xenografts
Source: PLoS One. 2018 Jun 12;13(6):e0199024. doi: 10.1371/journal.pone.0199024 (PMC5997322; doi:10.1371/journal.pone.0199024)

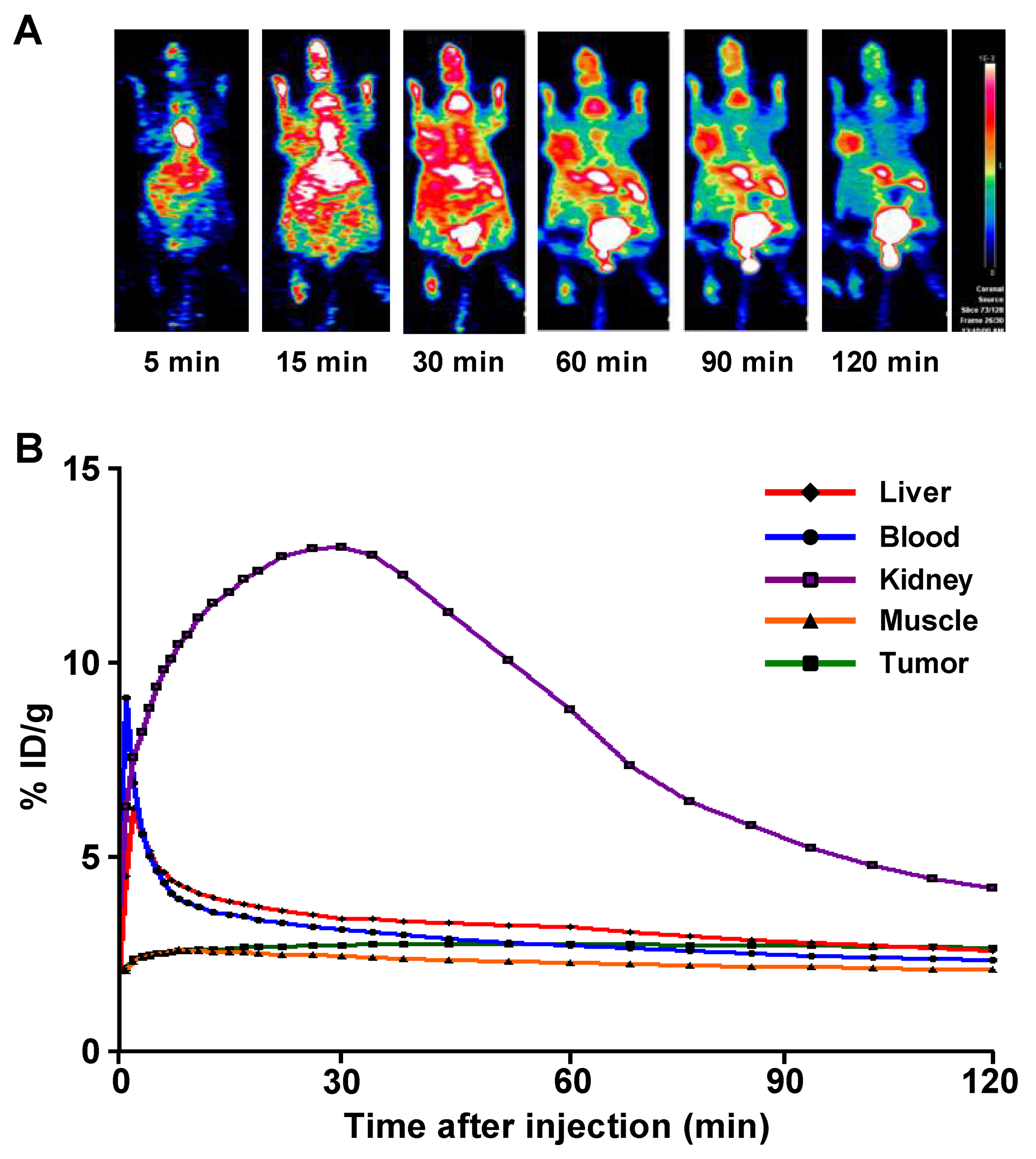

Supplement: S1 Fig — (A) Dynamic decay-corrected whole-body coronal microPET images after injection of 3.7 MBq (100 μCi) of [18F]FP-Met-pep1 in UM-SCC-22B tumor–bearing mice. (B) Time-activity curves of [18F]FP-Met-pep1 in UM-SCC-22B tumor, blood, liver, kidney and muscle. (TIFF) [file pone.0199024.s001.tiff]

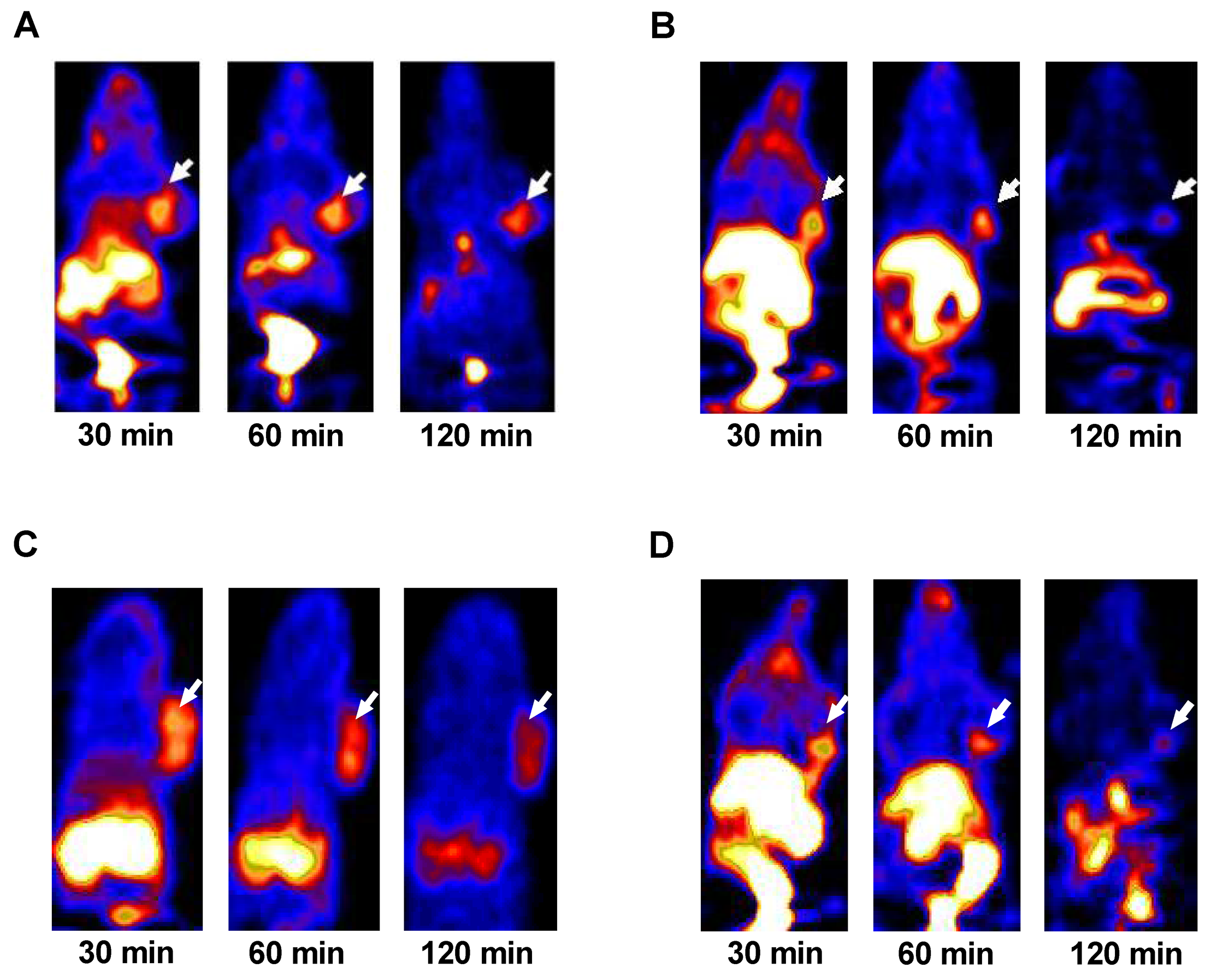

Supplement: S2 Fig — In vivo microPET images of four tumor-bearing mice (A)—(D) at 30, 60 and 120 min after injection of 3.7 MBq (100 μCi) of [18F]FP-Met-pep1. Tumors are indicated by arrows. (TIFF) [file pone.0199024.s002.tiff]
